# Age-Dependent Increase in Incidence of *Staphylococcus aureus* Bacteremia, Denmark, 2008–2015

**DOI:** 10.3201/eid2505.181733

**Published:** 2019-05

**Authors:** Louise Thorlacius-Ussing, Haakon Sandholdt, Anders Rhod Larsen, Andreas Petersen, Thomas Benfield

**Affiliations:** Hvidovre Hospital, University of Copenhagen, Copenhagen, Denmark (L. Thorlacius-Ussing, H. Sandholdt, T. Benfield);; Statens Serum Institut, Copenhagen (A. Larsen, A. Petersen)

**Keywords:** bacteremia, Staphylococcus aureus, incidence, population, epidemiology, bacteria, Denmark, aging, elderly, staphylococci

## Abstract

*Staphylococcus aureus* bacteremia (SAB) is a major cause of illness and death worldwide. We analyzed temporal trends of SAB incidence and death in Denmark during 2008–2015. SAB incidence increased 48%, from 20.76 to 30.37 per 100,000 person-years, during this period (p<0.001). The largest change in incidence was observed for persons >80 years of age: a 90% increase in the SAB rate (p<0.001). After adjusting for demographic changes, annual rates increased 4.0% (95% CI 3.0–5.0) for persons <80 years of age, 8.4% (95% CI 7.0–11.0) for persons 80–89 years of age, and 13.0% (95% CI 9.0–17.5) for persons >90 years of age. The 30-day case-fatality rate remained stable at 24%; crude population death rates increased by 53% during 2008–2015 (p<0.001). Specific causes and mechanisms for this rapid increase in SAB incidence among the elderly population remain to be clarified.

## Abstract 2

During 2008–2015, overall incidence increased by 50%, with a dramatic increase in persons >80 years of age.

*Staphylococcus aureus* is the most frequent gram-positive bacterium to cause invasive bloodstream infection (*1*). *S. aureus* bacteremia (SAB) is associated with considerable illness and death, yielding a case-fatality rate of 20%–25% (*2*). The occurrence of SAB has changed over time (*3*–*5*). Increasing incidence rates of SAB have been reported worldwide throughout the past few decades (*3*,*5*). However, more recent studies have reported stable or decreasing rates of SAB, as well as improved short-term death rates (*1*,*2*,*4*,*6*). As a result of demographic changes, including a rapidly increasing elderly population, contemporary analysis of the epidemiology of SAB is necessary to prioritize and allocate healthcare resources. Because concurrent conditions are more common with age, the changing demographics are of particular concern; older age and concurrent conditions are strongly associated with an increased risk for SAB (*2*,*4*,*7*).

Population-based studies are necessary to obtain valid epidemiologic data regarding SAB (*8*). Using surveillance data from the ongoing national registration of SAB in Denmark, we conducted a nationwide cohort study of temporal changes in SAB. The aims of this study were 2-fold. First, we analyzed temporal changes in SAB incidence; second, we assessed short-term death rates and associated risk factors.

## Methods

### Study Setting

We conducted a nationwide study of SAB in Denmark during 2008–2015. The population of Denmark comprised 5,475,791 residents in 2008 and 5,659,715 residents in 2015; all had free access to tax-financed healthcare. This study was approved by the Danish Data Protection Agency (approval nos. 2009-41-4179 and 2014-41-3376). Legislation in Denmark does not require informed consent for register-based studies.

### Study Population

We identified cases of SAB using data from the continuous national SAB surveillance in Denmark (*5*). Inclusion in the register was based on the identification of *S. aureus* in >1 blood culture. We defined cases in the study as those in patients with a first-time episode of SAB recorded during January 1, 2008–December 31, 2015.

### Data Sources

The unique civil registration number assigned to residents of Denmark by the Civil Registration System tracks information on vital and immigrant status and enables linkage of nationwide administrative healthcare registers on an individual level (*9*). The registry is updated daily.

The Danish National Patient Registry contains discharge diagnoses (from the International Classification of Diseases, 10th Revision) for residents regarding all hospital contacts (inpatient and outpatient) (*10*). The Danish National Bureau of Statistic provides data on the resident population and number of hospital admission and days in the hospital. The Danish Microbiology Database has conducted national surveillance on infectious diseases and microorganisms since 2010 (*11*).

### Variables of Interest

We analyzed and stratified age by 11 age groups: <1, 1–9; 10–19; 20–29; 30–39; 40–49; 50–59; 60–69; 70–79; 80–89 and >90 years. We used the Charlson Comorbidity Index (CCI) was used as a general measure of concurrent conditions; this index has previously been validated for SAB (*12*). We categorized CCI score into 3 levels: no concurrent conditions (CCI score = 0), intermediate (CCI score = 1–2), or high (CCI score >3) (*2*).

Recent hospital contact within 90 days before SAB was applied as a proxy of healthcare- and hospital-acquired SAB. We have previously validated this approach with a positive predictive value of 83% of distinguishing between healthcare/hospital- and community-associated SAB (*13*).

### Statistics

We reported counts by median and interquartile range (IQR). We computed crude incidence rates of SAB overall and by age, sex, and calendar year and calculated the incidence rates as the number of SAB cases per 100,000 person-years at risk and estimated person-years at risk assuming a uniform death rate throughout the year in the background population. We assessed temporal trends by comparing the incidence rate ratio (IRR) in 3 time periods, 2008–2010, 2011–2012, and 2013–2015. In addition, we used a Poisson regression model to evaluate the association of calendar year and incidence of SAB and added all available variables to the model hypothesized as confounders for the outcome in question. We validated the model assumptions using a quasi-Poisson model.

We performed several sensitivity analyses to assess whether changes in SAB incidence were associated with the rate of *S. aureus* isolates per 10,000 blood cultures, per 100,000 hospital admissions, or per 100,000 hospital days; changes in the relative difference of CCI score during 2008–2014 between cases and the population controls, by randomly matching 10 population controls by age and gender to each SAB case; and the proportion of SAB cases during 2008–2014 with hospital contact within 90 days before the diagnosis of SAB. We reported all-cause death rates for SAB cases as a 30-day case-fatality rate (CFR) and deaths as a percentage of the number of cases. In addition, we calculated crude population death rates as deaths of SAB cases per 100,000 person-years in the background population and applied a mortality rate ratio (MRR) to assess temporal trends in death rates. We used logistic regression models to identify any association between risk factors and 30-day death rates and adjusted for the following covariates: age, gender, calendar year, CCI score, and 90-day prior hospital contact. We present risk estimates as odds ratio (OR) with 95% CIs, and for all analyses, we considered a p value <0.05 to be significant. We performed statistical analyses using R software version 3.2.3 (R Project for Statistical Computing, https://www.r-project.org).

## Results

### Demographics

We identified a total of 11,054 incident cases of SAB during the study period. Demographics of the study population are provided in Table 1. In brief, the median age was 68 years (IQR 56–79 years); 62% of patients were male and 38% female. More than 75% of SAB case-patients had >1 concurrent condition recorded before the SAB episode. Methicillin-resistant *S. aureus* (MRSA) accounted for 1.3% of cases.

**Table 1:** Demographic characteristics of patients with *Staphylococcus aureus* bacteremia, Denmark, 2008–2015*

Characteristic	Value
Sex	
F	4,161 (37.6)
M	6,893 (62.4)
Age, y	
<1	282 (2.6)
1–9	157 (1.4)
10–19	208 (1.9)
20–29	204 (1.8)
30–39	374 (3.4)
40–49	792 (7.2)
50–59	1,326 (12.0)
60–69	2,486 (22.4)
70–79	2,561 (23.2)
80–89	2,121 (19.2)
>90	543 (4.9)
Median (IQR)	68 (56.0–79.0)
Mean (SD)	64 (63.9–64.7)
CCI score	
0	2,599 (23.5)
1–2	4,171 (37.7)
>3	4,284 (38.8)
MSSA	10,911 (98.7)
MRSA	143 (1.3)

*Values are no. (%) patients except as indicated. CCI, Charlson Comorbidity Index; IQR, interquartile range; MRSA, methicillin-resistant *S. aureus;* MSSA, methicillin-susceptible *S.* *aureus*.

### Incidence Rates

**Figure 1:**
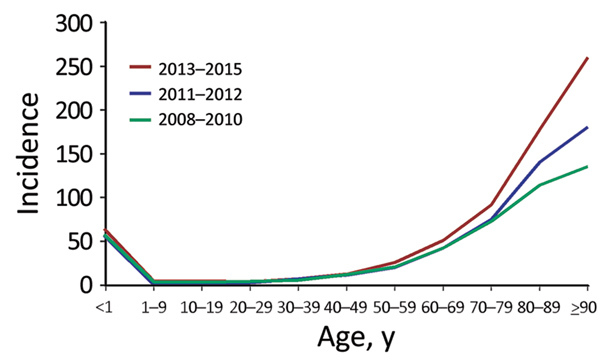
Temporal changes in *Staphylococcus aureus* bacteremia incidence (cases per 100,000 person-years), by age group and years, Denmark, 2008–2015.

The number of patients with SAB increased from 1,131 in 2008 to 1,731 in 2015, corresponding to a 48% increase in incidence, from 20.76 (95% CI 19.57–22.01) to 30.73 (95% CI 29.30–32.21) (IRR 1.48 [95% CI 1.37–1.59]), compared with an average annual incidence rate of 24.93 (95% CI, 24.47–25.40) cases/100,000 person-yeras. The highest incidence rates were observed among male patients, the elderly, and infants <1 year of age (Table 2; Figure 1; Appendix Tables 1–3).

**Table 2:** Incidence rate and incidence rate ratio of *Staphylococcus aureus* bacteremia stratified by sex and age, Denmark, 2008–2015*

Characteristic	IR, SAB/100.000 PY (95% CI)	IRR (95% CI)†	p value
Sex			
F	18.62 (18.06–19.19)	Referent	NA
M	31.35 (30.61–32.10)	2.00 (1.92–2.08)	<0.001
Age, y			
<1	57.96 (51.28–65.12)	17.37 (14.28–21.13)	<0.001
1–9	3.35 (2.84–3.91)	Referent	NA
10–19	3.75 (3.26–4.30)	1.12 (0.91–1.38)	0.281
20–29	3.84 (3.33–4.41)	1.15 (0.91–1.38)	0.199
30–39	6.50 (5.85–7.19)	1.96 (1.63–2.34)	<0.001
40–49	12.21 (11.37–13.09)	3.66 (3.09–4.34)	<0.001
50–59	22.92 (21.70–24.18)	6.88 (5.84–8.12)	<0.001
60–69	46.14 (44.35–47.99)	13.93 (11.86–16.36)	<0.001
70–79	81.26 (78.15–84.47)	24.93 (21.23–29.28)	<0.001
80–89	145.17 (139.05–151.45)	40.02 (33.35–48.03)	<0.001
>90	197.03 (180.80–214.32)	49.97 (39.19–63.72)	<0.001
Period, per-year increment	NA	1.04 (1.03–1.05)	<0.001

*IR, incidence rate; IRR, incidence rate ratio; NA, not applicable; PY, person-years; SAB, *Staphylococcus aureus* bacteremia. †Adjusted for sex, age, and period.

For persons ≥80 years of age, the incidence rate increased consistently throughout the 8-year study period (Figure 1). As such, during 2008–2015, the relative proportion of cases in persons >80 years of age increased from 19.72% (95% CI 17.40–22.04) to 26.17% (95% CI 24.10–28.24) of all cases. Further, the age-specific incidence rate increased significantly, by an estimated 56% (IRR 1.56 [95% CI 1.41–1.72]) for persons 80–89 years of age and 92% (IRR1.92 [95% CI 1.57–2.37]) for patients ≥90 years of age in 2013–2015 compared with 2008–2010. For persons <80 years of age, only rates for patients 50–79 years of age increased in 2013–2015 compared with 2008–2010 (50–59 years, IRR 1.24 [95% CI 1.09–1.40]; 60–69 years, IRR 1.20 [95% CI 1.10–1.32]; 70–79 years, IRR 1.26 [95% CI 1.15–1.38]) (Appendix Table 1). For 2011–2012, the incidence rate for persons <80 years of age did not differ compared with the rates for 2008–2010. Gender did not affect the age-specific trends in SAB rate (Appendix Tables 2, 3).

**Figure 2:**
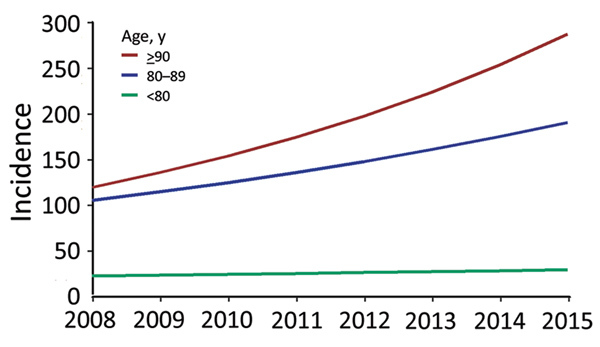
Increase in incidence of *Staphylococcus aureus* bacteremia for persons >80 years of age compared with younger persons, Denmark, 2008–2015.

Regression analysis indicated that age was strongly associated with SAB incidence (Table 2). Additionally, male case-patients had a 2-fold higher risk (IRR 2.00 [95% CI 1.92–2.08]) of acquiring SAB than female case-patients. After adjustment for demographic changes, the estimated annual rates of SAB increased by 4% (IRR 1.04 [95% CI 1.03–1.05]) for persons <80 years of age, 8% (IRR 1.08 [95% CI 1.07–1.11]) for persons 80–89 years of age, and 13% (IRR 1.13 [95% CI 9.0–17.5]) for persons >90 years (Figure 2; Appendix Figure 1).

### Blood Culture Activity, Hospital Admission, and Hospital Stays

The number of blood cultures performed in the healthcare system in Denmark increased from 367,884 in 2010 to 480,892 in 2015. The positivity rate of *S. aureus* per 10,000 blood cultures increased from 33.43 (95% CI 31.59–35.36) to 36.00 (95% CI 34.32–37.73) during that period, corresponding to an increase of 8% (95% CI 0%–16%) (Appendix Table 4). The rate of SAB cases per 10,000 blood cultures remained stable for all age groups across the study period (Appendix Figure 2, Table 5). Annual hospital admissions increased from 1,175,452 to 1,347,563, but the total number of hospital days admitted decreased from 4,854,060 to 4,067,222 during 2008–2015. After adjustment, the number of SAB cases per 100,000 hospital admissions increased by 33% (95% CI 24%–44%) and SAB cases per 100,000 hospital days increased by 83% (95% CI 69%−97%) (Appendix Table 4).

### Concurrent Conditions and Recent Hospital Contact before SAB

Patients with SAB had significantly more concurrent conditions than the matched population controls (mean CCI 2.43 for case-patients compared with 0.81 for population controls; p<0.001). SAB case-patients and population controls both had increasingly more concurrent conditions during the study period, but the changes over time were similar for case-patients and population controls overall and stratified by age (Appendix Figure 3). Approximately 75% (n = 6,707) of case-patients had had hospital contact within 90 days before their SAB diagnosis. The proportion of SAB cases with recent hospital contact remained unchanged over the years (Appendix Figure 4, panel A). Stratification by age did not affect this result (Appendix Figure 4, panel B).

### CFR, Population Death Rate, and Associated Risk Factors

**Figure 3:**
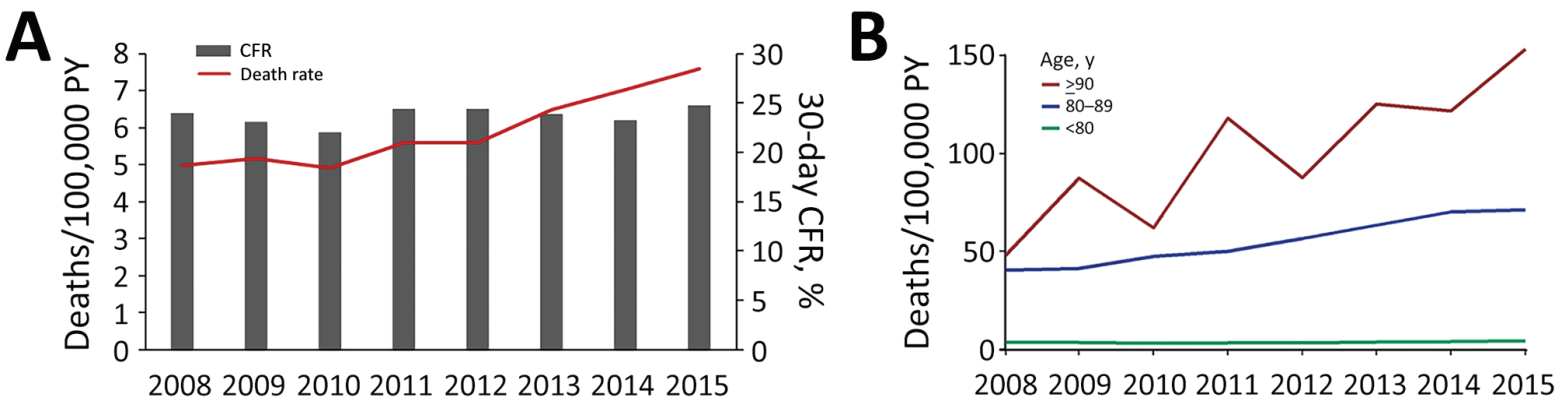
*Staphylococcus aureus* bacteremia deaths, Denmark, 2008–2015. A) Overall population death rate and 30-day CFR. B) Population death rates for persons >80 years of age compared with younger persons. PY, person-years.

In the 8-year study period, 30-day all-cause deaths increased from 271 in 2008 to 428 in 2015. The population death rate for SAB rose significantly, from 4.97 (95% CI 4.40–5.60) to 7.60 (95% CI 6.90–8.35), corresponding to an estimated increase of 53% (MRR 1.53 [95% CI 1.31–1.78]) (Figure 3, panel A). Stratification by age group showed great variation in trends of the age-specific death rate, with the most rapid increase in death rate for the oldest age groups (Figure 3, panel B). For persons <80 years of age, the death rate increased by 25% (MRR 1.25 [95% CI 1.03−1.51]).

The overall 30-day CFR for the study period was 24% (95% CI 23%–25%) and remained unchanged over the years (Table 3; Figure 3, panel A). A higher 30-day CFR was observed with increasing age and CCI score, and for female compared with male case-patients (Table 3; Appendix Table 6). The 30-day CFR did not differ over time by gender or age (Appendix Figures 5, 6). Multivariate analysis indicated that age was strongly associated with 30-day CFR (Table 3). Any concurrent condition before SAB was also associated with higher risk of death. Compared with persons with no concurrent conditions, the risk for death increased for persons with increasing CCI scores (OR 1.32 [95% CI 1.14–1.55] for CCI score 1–2 and OR 1.67 [95% CI 1.43–1.94] for CCI score >3). Female sex was an independent risk factor for death compared with male sex (OR 1.20 [95% CI 1.08–1.33]). In contrast, prior hospital contact and time period were not associated with 30-day CFR.

**Table 3:** CFR and associated risk of *Staphylococcus aureus* bacteremia, Denmark, 2008–2015*

Characteristic	30-d CFR (95% CI)	Multivariate OR (CI 95%)	p value
Sex			
M	21.88 (20.79–23.01)	Referent	NA
F	26.92 (25.36–28.54)	1.20 (1.08–1.33)	<0.001
Age, y			
<1	6.74 (4.06–10.52)	4.68 (1.05–20.85)	0.043
1–9	2.55 (0.69–6.52)	Referent	NA
10–19	1.92 (0.52–4.92)	1.71 (0.31–9.50)	0.538
20–29	2.94 (1.08–6.40)	2.22 (0.44–11.20)	0.333
30–39	2.13 (0.92–4.21)	1.36 (0.28–6.64)	0.705
40–49	9.47 (7.45–11.87)	6.37 (1.54–26.34)	0.011
50–59	16.44 (14.33–18.77)	10.79 (2.64–44.06)	<0.001
60–69	19.79 (18.08–21.62)	13.00 (3.19–52.89)	<0.001
70–79	27.80 (25.80–29.92)	19.69 (4.85–79.94)	<0.001
80–89	38.10 (35.51–40.82)	32.26 (7.94–131.03)	<0.001
>90	51.93 (46.05–58.36)	58.48 (14.26–239.82	<0.001
CCI score			
0	14.20 (12.79–15.72)	Referent	NA
1–2	23.90 (22.44–25.43)	1.32 (1.14–1.55)	<0.001
>3	29.46 (27.86–31.13)	1.67 (1.43–1.94)	<0.001
Hospital contact within 90 d			
No	22.21 (20.34–24.23)	Referent	NA
Yes	23.98 (22.82–25.18)	1.10 (0.97–1.24)	0.130
Period, per-year increment	NA	0.99 (0.97–1.01)	0.044

*CCI, Charlson Comorbidity Index; CFR, case-fatality rate; NA, not applicable; OR, odds ratio.

## Discussion

In this nationwide study, we evaluated trends of SAB incidence in the population of Denmark in an 8-year period, corresponding to >40 million patient-years. We report a 48% increase in SAB incidence during 2008–2015. Although the short-term death rate remained unchanged throughout the period, population-based death rates increased more than 50% because of the increase in SAB incidence.

The reported increase in SAB incidence was, in particular, a result of the major and consistent increase among persons >80 years of age: the SAB rate among the oldest old (>80 years) rose with an alarming 90% during 2008–2015, corresponding to an estimated annual increase of 8%–13% in incidence rate. In comparison, a 36% increase in the incidence rate was found for persons <80 years of age.

The differential increase in the SAB rate among age groups stresses the importance of comprehensive data that enable age-stratified analyses when investigating temporal trends in infectious diseases. Significant changes in death and incidence rates within subgroups of a population may otherwise not be apparent.

The increasing incidence rate observed in this study is contrary to most other recent studies. Stable or decreasing rates of SAB were reported in several large observational studies (*2*,*4*–*6*,*14*,*15*). However, increasing rates of both community- and healthcare-acquired SAB were reported in a recent study in Finland (*16*). A higher occurrence of SAB is somewhat to be expected in an increasingly elderly population. However, our data showed an increase in SAB occurrence that exceeded contemporary changes in the population demographic profile.

Multiple factors may have influenced the changes in SAB incidence. Concurrent conditions form a strong risk factor associated with development of SAB; the increasing burden of concurrent conditions associated with aging could explain the higher SAB rate among the oldest elderly (*2*). The study population generally developed more concurrent conditions during the study period; thus, the number of persons at risk of acquiring SAB increased. However, because concurrent conditions increased similarly for SAB cases and population controls, the increasing incidence of SAB could not be explained solely by an increase in these conditions.

A more liberal use of invasive hospital procedures and immune modulating treatments may also contribute to a higher occurrence of SAB. Although the proportion of SAB cases with 90-day prior hospital contact remained unchanged throughout the study, we do not know whether rates of invasive procedures and immune-suppressive medication increased in the same period. Thus, rates of hospital contacts might not be an accurate measure; recent reports showed an increasing use of chemotherapy and invasive procedures over time, in particular among the elderly (*17*,*18*).

The average life expectancy in Denmark increased during the study period, leading to an increasingly older and potentially more fragile population (*19*). There may be biologic risk factors of infectious diseases associated with aging that have not yet been identified. Physiologic age-dependent changes, such as immunological senescence, are likely attributable, in part, to the higher vulnerability to infectious diseases among elderly persons (*20*,*21*).

Higher rates of hospitalization and test activity and longer in-hospital stays may be other potential explanations for the increasing SAB rate (*22*). Blood culture activity increased by 31% during the study period but the increase in the SAB positivity rate was 8%, suggesting that the increase in activity alone could not explain the observed changes. Further, a systematic increase in culture activity or higher sensitivity of culture systems would not explain a differential increase in incidence among age groups. Higher test rates would likely lead to the identification of milder cases of SAB. If so, a contemporary decline in the overall 30-day CFR would be expected, given higher survival rates among the less severe cases, but this was not the case.

Annual hospital admission rates increased by 15% and the number of days hospitalized declined by 16% during the study period, suggesting that changes in hospitalizations did not explain the increased rates of SAB. The increase in rates of SAB remained after adjustment for hospitalizations and hospital days. An increase in hospital-acquired infections is an unlikely explanation for the increased SAB rates because the proportion of SAB patients with and without 90-day prior hospital contact remained unchanged throughout the study.

A recent study found that the increase in sepsis rates and decline in death rates in California were associated with up-capture of less severely ill patients after introduction of guidance on coding based on the International Classification of Diseases, Ninth Revision (*23*). Our study, however, relied on blood culture positive cases of SAB; therefore, changes in coding practices are an unlikely explanation of our finding. Further, death rates were unchanged over time in our study.

In spite of a recent report of a decline in death associated with infectious diseases in general, SAB survival has not improved markedly during the past decades (*24*). The 30-day CFR of 24% found in our study is in line with other reports (*25*–*27*). However, the population death rate rose 1.5-fold, to 7.60/100,000 person-years by 2015, because of the contemporary increase in SAB incidence. Thus, population death rates may represent a more accurate measure of the actual disease burden, as changes in incidence are reflected despite an unchanged 30-day CFR. Few studies have reported SAB death rates as population death rates for comparison and none have reported age-specific death rates (*28*,*29*). Tom et al. reported a death rate from community-acquired SAB of 3.4/100,000 person-years (*28*). A recent study from Norway on bloodstream infections found a death rate for SAB similar to ours, of 7/100,000 person-years (*29*).

A particular finding in our study was the close association between the changes in the overall death rate and the increase in the age-specific death rate for the oldest age groups. The higher incidence rates for the oldest age groups were directly reflected by higher population death rates. Death rates from SAB will likely increase even more over the next decades owing to the increasingly older population, which may also be the case for other invasive bloodstream infections. However, temporal population death rates from nonstaphylococcal bloodstream infections have not been reported; further studies are warranted to address this matter.

Contrary to the case with most bloodstream infections, female sex has been associated with an inferior outcome in SAB (*25*). In agreement with several previous observations, we found a significantly higher death rate among women compared with men (*7*,*25*,*30*,*31*). The mechanisms underlying the observed gender differences in SAB death rates are not fully understood; a recent study did not find any gender-specific differences in clinical management, patient characteristics, or severity of the disease between men and women (*32*).

This study benefits from the large number of observational years, nationwide settings, and standardized registration. Nonetheless, some limitations must be noted. First, the increasing life expectancy during the study period could have led to an increasing median age within age groups and, consequently, an underestimation of the effect of age on incidence rates, in particular for the oldest persons. Second, the SAB definition was based solely on microbiological findings and not related to criteria of clinical infection such as systemic inflammatory response syndrome and sequential organ failure assessment score. Thus, we cannot preclude that a minor proportion of the positive blood cultures may be from contamination rather than clinical infection. Still, this possibility does not explain the age-dependent increase in SAB. Third, we were not able to stratify SAB origin by community-, healthcare-, and hospital-acquired infections, because these data were not accessible. Instead, we used a validated approach to access temporal changes in healthcare/hospital- and community-acquired infections. Fourth, data on blood culture activity were not available for 2008–2009; thus, we were not able to analyze trends in culture rates for the whole study period. Finally, we did not have access to clinical data regarding the primary focus of the infection, the severity of the disease, and the applied treatment strategies, as well as the effect of these potential risk factors on SAB death.

MRSA bacteremia is infrequent in Denmark (1.3% of all SAB cases), which could theoretically limit the validity of the results of this study to settings with higher MRSA prevalence. Nevertheless, in most populations, methicillin-susceptible *S. aureus* (MSSA) bacteremia has remained prevalent despite the emergence of MRSA and, as such, several studies have concluded that MRSA bacteremia adds to the total burden of SAB rather than replacing MSSA bacteremia (*16*,*33*). Thus, we believe that the observed changes in MSSA bacteremia in our study may be applicable to other populations.

In conclusion, SAB incidence in Denmark increased by 48% during 2008–2015. SAB rates increased the most among the oldest age group, for whom the age-specific incidence rate nearly doubled and where increases in rates exceeded the contemporary changes in the population demographic profile. Furthermore, the short-term prognosis of SAB did not improve within the study period and, combined with the increasing incidence, population death rates rose significantly. Our results stress that infection prevention initiatives and improved care are warranted to reduce SAB incidence and improve outcomes. In addition, examinations of the burden of bloodstream infections caused by MSSA must be prioritized in future research; a specific focus should be on the frail elderly population.

Appendix Additional information on study of age-dependent increase in *Staphylococcus aureus* bacteremia, Denmark, 2008–2015.
