# Supplementary material for: Age-Dependent Increase in Incidence of Staphylococcus aureus Bacteremia, Denmark, 2008–2015
Source: Emerg Infect Dis. 2019 May;25(5):875–82. doi: 10.3201/eid2505.181733 (PMC6478196; doi:10.3201/eid2505.181733)
Supplement: Appendix — Additional information on study of age-dependent increase in Staphylococcus aureus bacteremia, Denmark, 2008–2015. [file 18-1733-Techapp-s1.pdf]

Article DOI: DOI: https://doi.org/10.3201/eid2505.181733

Age-Dependent Increase in Incidence of *Staphylococcus aureus* Bacteremia, Denmark, 2008–2015

Appendix

Appendix Table 1. Incidence rates and incidence rate ratios of *Staphylococcus aureus* bacteremia in Denmark, 2008–2015, stratified by age group

	Incidence rate of *Staphylococcus aureus* bacteremia per 100.000 person-years (95% CI)										
Age, y	<0	1–9	10–19	20–29	30–39	40–49	50–59	60–69	70–79	80–89	>90
Year											
2008	57.56 (40.53–79.34)	4.55 (3.00–6.62)	2.04 (1.12–3.42)	4.01 (2.60–5.92)	4.77 (3.34–6.61)	11.89 (9.63–14.52)	20.53 (17.35–24.13)	41.79 (36.92–47.12)	73.20 (64.57–82.66)	107.30 (92.69–123.55)	95.24 (64.25–135.95)
2009	53.67 (37.39–74.65)	3.54 (2.19–5.42)	4.47 (3.04–6.35)	4.76 (3.21–6.79)	6.28 (4.62–8.36)	11.20 (9.02–13.75)	20.77 (17.56–24.40)	44.96 (39.97–39.97)	70.67 (62.28–79.88)	123.22 (107.57–140.49)	156.25 (115.97–205.99)
2010	57.08 (39.98–79.02)	3.54 (2.19–5.42)	3.88 (2.56–5.64)	4.08 (2.66–5.98)	6.08 (4.43–8.13)	13.15 (10.78–15.89)	22.04 (18.73–25.77)	41.66 (36.91–46.86)	75.10 (66.53–84.47)	112.96 (98.02–129.52)	155.16 (115.16–204.55)
2011	53.43 (37.00–74.66)	0.68 (0.18–1.74)	3.15 (1.97–4.77)	2.31 (1.29–3.81)	7.51 (5.66–9.78)	13.54 (11.13–16.32)	21.68 (18.40–25.38)	40.82 (36.15–45.92)	77.83 (69.21–87.22)	131.30 (115.14–149.08)	185.92 (142.87–237.88)
2012	55.74 (38.37–78.27)	0.68 (0.19–1.74)	2.44 (1.42–3.90)	3.62 (2.32–5.38)	6.57 (4.82–8.73)	9.70 (7.68–12.09)	19.73 (16.62–23.26)	44.71 (39.85–50.01)	72.50 (64.31–81.44)	149.73 (132.46–168.62)	175.59 (134.62–225.09)
2013	70.51 (50.60–95.65)	5.31 (3.61–7.53)	3.32 (2.10–4.98)	3.81 (2.49–5.59)	5.15 (3.60–7.12)	12.51 (10.20–15.19)	25.07 (21.56–29.99)	46.27 (41.33–51.63)	85.55 (77.77–96.05)	164.94 (146.82–184.63)	269.99 (218.95–329.37)
2014	58.88 (40.53–82.69)	4.15 (2.66–6.17)	5.21 (3.65–7.22)	4.56 (3.12–6.44)	6.98 (5.15–9.26)	12.98 (10.61–15.71)	26.91 (23.29–30.93)	55.05 (49.66–60.88)	93.39 (84.47–102.99)	185.54 (166.42–206.25)	224.91 (179.14–278.81)
2015	57.87 (38.84–81.27)	4.37 (2.83–6.45)	5.54 (3.92–7.60)	3.59 (2.35–5.27)	8.84 (9.75–11.38)	12.71 (10.36–15.43)	26.30 (22.75–30.23)	53.30 (47.97–59.06)	95.27 (86.49–104.69)	183.65 (164.78–204.09)	285.43 (234.14–344.61)
Incidence rate ratio (95% CI)											
Period											
2008–2010	1	1	1	1	1	1	1	1	1	1	1
2011–2012	0.18 (0.06–0.33)	0.97 (0.71–1.31)	0.81 (0.53–1.17)	0.69 (0.46–1.00)	1.23 (0.94–1.60)	0.96 (0.80–1.15)	0.98 (0.88–1.14)	1.00 (0.90–1.11)	1.03 (0.92–1.15)	1.23 (1.09–1.38)	1.33 (1.04–1.71)
2013–2015	1.19 (0.86–1.67)	1.11 (0.86–1.47)	1.35 (1.00–1.86)	0.93 (0.68–1.27)	1.22 (0.96–1.55)	1.05 (0.90–1.24)	1.24 (1.09–1.40)	1.20 (1.10–1.32)	1.26 (1.15–1.38)	1.56 (1.41–1.72)	1.92 (1.57–2.37)

Appendix Table 2. Incidence rates and incidence rate ratios of *Staphylococcus aureus* bacteremia among female persons in Denmark, 2008–2015, stratified by age group

Age	<0	1–9	10–19	20–29	30–39	40–49	50–59	60–69	70–79	80–89	>90
	Incidence rate of *Staphylococcus aureus* bacteremia per 100,000 person-years (95% CI)										
Year											
2008	60.55 (36.46–94.56)	4.14 (2.14–7.23)	2.10 (0.84–4.32)	1.94 (0.71–4.23	4.54 (2.65–7.28)	10.05 (7.18–13.69)	13.43 (9.90–17.81)	28.82 (23.26–35.31)	48.86 (39.53–59.73)	70.73 (56.17–87.91)	66.82 (38.19–108.52)
2009	56.96 (33.76–90.01)	3.45 (1.66–6.35)	3.26 (1.63–5.83)	3.85 (1.99–6.73)	5.11 (3.08–7.97)	8.49 (5.88–11.87)	16.59 (12.63–21.40)	27.17 (21.85–33.39)	45.61 (36.68–56.06)	81.29 (65.51–99.59)	107.24 (70.05–157.13)
2010	61.90 (37.27–96.97)	3.08 (1.90–6.80)	2.06 (0.83–4.25)	3.49 (1.74–6.25)	5.42 (3.31–8.37)	9.72 (6.91–13.29)	14.06 (10.44–18.54)	24.53 (19.54–30.41)	52.32 (42.79–63.33)	83.44 (67.51–102.00)	114.97 (76.40–116.17)
2011	48.29 (27.03–79.65)	0.35 (0.01–1.94)	1.76 (0.65–3.84)	2.18 (0.88–4.50)	6.30 (3.99–9.45)	6.98 (4.64–10.09)	12.90 (9.44–17.20)	24.98 (19.98–30.85)	54.55 (44.92–65.63)	98.43 (80.97–118.53)	133.34 (32.34–186.33)
2012	48.15 (26.33–80.79)	0.00 (0.00–1.29)	0.88 (0.18–2.58)	3.67 (1.89–6.40)	4.75 (2.77–7.61)	6.46 (4.22–9.47)	12.53 (9.14–16.76)	26.99 (21.81–33.03)	48.89 (39.91–59.30)	107.10 (88.79–128.06)	147.35 (104.78–201.43)
2013	67.36 (40.56–105.20)	5.62 (3.21–9.12)	2.66 (1.22–5.05)	2.98 (1.43–5.47)	3.72 (1.98–6.37)	7.43 (5.01–10.61)	18.49 (14.33–23.49)	29.43 (24.02–35.69)	50.73 (41.73–61.10)	115.80 (96.75–137.50)	212.52 (160.96–275.35)
2014	25.58 (10.29–52.71)	2.84 (1.22–5.59)	2.97 (1.42–5.46)	5.50 (3.31–8.60)	5.83 (3.56–9.01)	7.73 (5.25–10.97)	19.88 (15.58–25.00)	35.10 (29.18–41.88)	68.56 (58.26–80.17)	130.79 (110.50–153.72)	160.13 (116.35–214.97)
2015	57.75 (33.01–93.78)	2.51 (1.01–5.17)	4.18 (2.29–7.02)	2.25 (0.97–4.44)	6.53 (4.09–9.88)	9.79 (6.96–13.38)	17.68 (13.67–22.49)	28.18 (22.87–34.33)	63.51 (53.85–74.41)	143.01 (121.84–166.81)	235.33 (182.01–299.40)
	Incidence rate ratio (95% CI)										
Period											
2008–2010	1	1	1	1	1	1	1	1	1	1	1
2011–2012	0.81 (0.49–1.25)	0.05 (0.00–0.17)	0.53 (0.20–1.08)	0.95 (0.51–1.67)	1.10 (0.73–1.65)	0.71 (0.51–0.98)	0.87 (0.66–1.11)	0.97 (0.80–1.17)	1.06 (0.88–1.26)	1.31 (1.10–1.56)	1.46 (1.05–2.03)
2013–2015	0.84 (0.55–1.26)	0.96 (0.58–1.59)	1.32 (0.78–2.32)	1.15 (0.70–1.91)	1.06 (0.73–1.56)	0.88 (0.68–1.15)	1.27 (1.04–1.57)	1.15 (0,98–1.35)	1.25 (1.08–1.46)	1.66 (1.42–1.93)	2.10 (1.60–2.83)

Appendix Table 3. Incidence rates and incidence rate ratios of *Staphylococcus aureus* bacteremia among male persons in Denmark, 2008–2015, stratified by age group

	Incidence rate of *Staphylococcus aureus* bacteremia per 100.000 person-years (95% CI)										
Age	<0	1–9	10–19	20–29	30–39	40–49	50–59	60–69	70–79	80–89	>90
Year											
2008	54.71 (32.42–86.46)	4.94 (2.76–8.14)	1.99 (0.80–4.10)	6.04 (3.63–9.43)	5.00 (3.01–7.81)	13.68 (10.33–17.76)	27.60 (22.43–33.60)	55.12 (47.21–63.97)	102.63 (87.57–119.55)	171.37 (141.10–206.20)	185.26 (101.28–310.86)
2009	50.59 (29.47–81.00)	3.63 (1.81–6.49)	5.63 (3.44–8.69)	5.64 (3.34–8.92)	7.44 (4.94–10.75)	13.83 (10.48–17.92)	24.93 (20.02–30.68)	63.24 (54.86–72.54)	63.24 (54.86–72.54)	195.25 (163.13–231.85)	309.42 (198.25–460.4)
2010	52.51 (30.59–84.07)	3.30 (1.58–6.06)	5.60 (3.42–8.65)	4.65 (2.61–7.68)	6.73 (4.35–9.93)	16.49 (12.80–20.90)	30.00 (24.58–36.25)	59.28 (51.25–68.21)	102.08 (87.44–118.48)	162.64 (133.67–169.03)	279.47 (175.14–423.12)
2011	58.33 (35.12–91.09)	0.99 (0.20–2.90)	4.47 (2.56–7.26)	2.44 (1.05–4.80)	8.72 (5.69–12.31)	19.94 (15.86–24.75)	30.43 (24.98–76.70)	57.12 (49.31–65.82)	105.14 (90.50–121.48)	185.42 (154.57–220.61)	345.79 (231.58–496.62)
2012	63.05 (37.96–98.47)	1.33 (0.36–3.39)	3.92 (2.14–6.57)	3.57 (1.84–6.24)	8.37 (5.65–11.95)	12.87 (9.64–16.84)	26.91 (21.82–32.83)	62.65 (54.78–72.00)	99.97 (85.93–115.64)	218.35 (185.02–255.95)	260.09 (164.88–390.27)
2013	73.47 (46.04–11.24)	5.01 (2.81–8.27)	3.94 (2.15–6.61)	4.63 (2.65–7.52)	6.56 (4.16–9.84)	17.49 (13.69–22.03)	31.62 (26.11–37.96)	63.62 (55.42–72.70)	127.76 (112.20–144.87)	242.84 (207.91–281.97)	439.25 (313.80–598.13)
2014	90.64 (59.21–132.81)	5.39 (3.08–8.76)	7.35 (4.80–10.78)	3.64 (1.94–6.23)	8.12 (5.40–11.74)	18.14 (14.24–22.77)	33.91 (28.22–40.40)	75.62 (66.65–84.45)	121.76 (106.96–138.03)	270.45 (233.91–311.07)	413.75 (294.21–565.61)
2015	57.99 (33.78–92.84)	6.13 (3.64–9.70)	6.83 (4.37–10.16)	4.89 (2.90–7.72)	11.13 (7.88–15.28)	15.59 (11.98–19.95)	34.85 (29.14–41.36)	79.26 (70.04–89.37)	131.25 (116.30–147.59)	245.39 (211.12–283.63)	428.90 (309.11–579.75)
	Incidence rate ratio (95% CI)										
Period											
2008–2010	1	1	1	1	1	1	1	1	1	1	1
2011–2012	1.15 (0.74–1.73)	0.29 (0.10–0.59)	0.95 (0.59–1.49)	0.55 (0.31–0.90)	1.34 (0.96–1.88)	1.12 (0.90–1.39)	1.04 (0.87–1.24)	1.01 (0.89–1.15)	1.01 (0.88–1.15)	1.15 (0.98–1.34)	1.17 (0.80–1.70)
2013–2015	1.41 (0.97–2.04)	1.39 (0.91–2.18)	1.37 (0.94–2.01)	0.81 (0.54–1.20)	1.34 (0.99–1.85)	1.16 (0.95–1.42)	1.22 (1.05–1.42)	1.23 (1.10–1.37)	1.25 (1.12–1.40)	1.43 (1.26–1.64)	1.65 (1.22–2.29)

Appendix Table 4. Rate of *Staphylococcus aureus* bacteremia (SAB) by blood culture activity, hospital admissions, and hospital days in Denmark, 2008–2015

Year	SAB cases	SAB/ 10,000 blood cultures	Hospitalizations	SAB/100,000 hospitalizations	Hospital days	SAB/100,000 hospital days
2008	1,131	NA*	1,175,452	96.22 (90.69–101.99)	4,854,060	23.30 (21.96–24.70)
2009	1,226	NA	1,246,896	98.32 (92.90–103.99)	4,765,003	25.73 (24.31–27.21)
2010	1,230	33.43 (31.59–35.36)	1,288,098	95.49 (90.23–100.98)	4,536,799	27.11 (25.62–28.67)
2011	1,269	34.53 (32.65–36.48)	1,294,073	98.06 (92.74–103.61)	4,376,628	28.99 (27.42–30.64)
2012	1,271	31.84 (30.11–33.63)	1,326,235	95.84 (90.64–101.25)	4,345,788	29.25 (27.66–30.90
2013	1,513	36.21 (34.40–38.08)	1,328,860	113.86 (108.19–119.74)	4,269,734	35.44 (33.67–37.27)
2014	1,683	35.83 (34.14–37.59)	1,363,202	123.46 (117.63–129.50)	4,312,451	39.03 (37.18–40.94)
2015	1,731	36.00 (34.32–37.73)	1,347,563	128.45 (122.47–134.65)	4,067,222	42.56 (40.58–44.61)

*NA, not applicable

Appendix Table 5. Rate of *Staphylococcus aureus* bacteremia by blood culture activity stratified by age groups, Denmark, 2010–2015

	Staphylococcus aureus bacteremia/10,000 blood cultures (95% CI)										
Age	<1	1–9	10–19	20–29	30–39	40–49	50–59	60–69	70–79	80–89	>90
Year											
2010	58.2 (40.8–80.6)	19.2 (11.8–29.3)	28.6 (18.9- 41.7)	17.9 (11.7–26.2)	22.6 (16.5–30.3)	37.0 (30.3–44.7)	34.2 (29.0–40.0)	35.2 (31.7–40.2)	34.4 (30.5–38.7)	34.4 (29.5–38.9)	37.7 (28.0–49.7)
2011	56.0 (38.8–78.2)	3.77 (1.03–9.66)	24.9 (15.6–37.7)	10.2 (5.7–16.8)	26.3 (19.8–34.2)	38.6 (31.7–46.5)	33.3 (28.3–39.0)	36.6 (32.6–41.0)	36.7 (32.7–41.2)	40.1 (35.2–45.5)	44.5 (34.2–56.9)
2012	54.4 (37.4 –76.4)	3.98 (1.08- 10.2)	20.5 (12.0–32.9)	15.5 (9.91–23.0)	23.7 (17.4–31.6)	26.0 (20.6–32.4)	29.6 (25.0–34.9)	36.6 (32.6–41.0)	31.2 (27.7–35.0)	39.1 (34.6–44.1)	36.2 (27.8–46.5)
2013	69.2 (49.6–93.8)	30.3 (20.6–43.0)	28.9 (18.5–43.0)	16.5 (10.8–24.1)	18.8 (13.2–26.0)	33.0 (26.9–40.1)	36.8 (31.7–42.6)	36.6 (32.4–40.5)	36.7 (33.0–40.8)	40.7 (36.2–45.5)	50.5 (41.0–61.6)
2014	53.9 (37.1–75.7)	24.6 (15.8–36.6)	35.1 (24.6–48.6)	17.0 (11.6–23.9)	22.6 (16.7–30.0)	31.6 (25.8–38.2)	35.6 (30.8–41.0)	39.1 (25.3–43.5)	35.9 (32.5–39.6)	40.8 (36.6–45.3)	37.9 (30.2–47.0)
2015	57.9 (39.9–81.3)	29.0 (18.7–42.8)	40.3 (28.5–55.0)	13.3 (8.67–19.4)	27.2 (20.8–35.0)	30.3 (24.7–36.8)	35.2 (30.4–40.5)	37.6 (33.9–41.7)	36.5 (33.1–40.1)	39.9 (35.8–44.4)	45.0 (36.9–54.4)

Appendix Table 6. Thirty-day case fatality rate (CFR) among *Staphylococcus aureus* bacteremia patients stratified by age group and Charlson Comorbidity Index (CCI) score*

	30-d CFR (95% CI)		
Age group, years	CCI score 0	CCI score 1–2	CCI score ≥3
<1	7.01 (4.22–10.95)	NA	NA
1–9	2.04 (0.25–7.37)	3.92 (0.47–14.17)	NA
10–19	1.89 (0.39–5.51)	2.50 (0.06–13.93)	NA
20–29	3.64 (0.99–9.31)	1.45 (0.04–8.07)	4.00 (0.10–22.29)
30–39	1.35 (0.16–4.88)	2.14 (0.44–6.26)	3.49 (0.72–10.19)
40–49	2.46 (0.90–5.35)	9.74 (6.57–13.90)	16.25 (11.56–22.21)
50–59	12.09 (8.68–16.41)	14.06 (10–98–17.73)	21.99 (18.01–26.60)
60–69	12.30 (9.21–16.08)	17.08 (14.53–19.95)	24.91 (22.08–28.01)
70–79	20.24 (15.69–25.71)	26.33 (23.15–29.82)	30.82 (27.87–34.01)
80–89	32.95 (27.23–39.53)	38.16 (34.27–42.38)	40.14 (36.00–44.63)
≥90	48.28 (36.47–62.69)	50.97 (42.64–60.44)	55.95 (45.23–68.47)

*CCI, Charlson Comorbidity Index; CFR, case fatality rate; NA, not applicable.

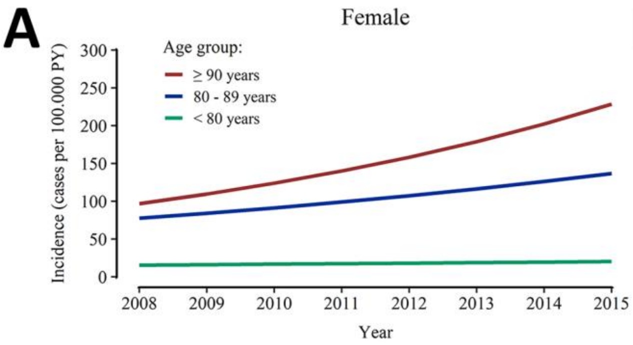

Appendix Figure 1. Estimated incidence of *Staphylococcus aureus* bacteremia (SAB) for A) females and B) males, Denmark, 2008–2015, by Poisson regression model.

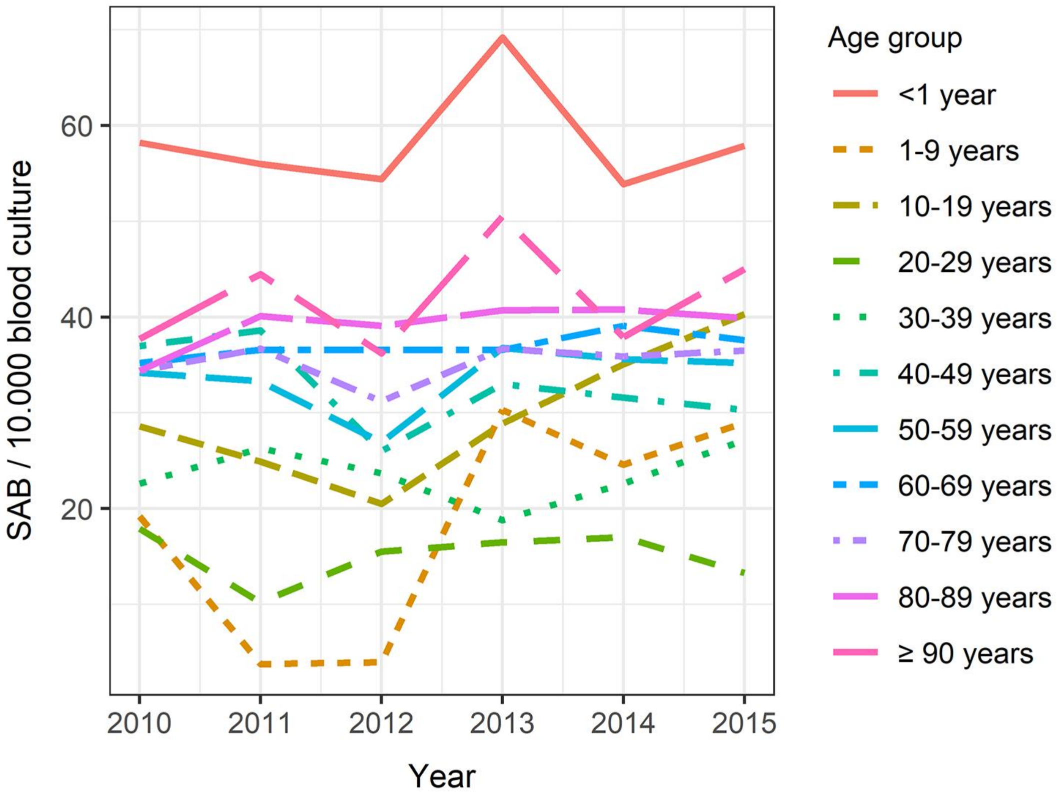

Appendix Figure 2. Plot depicting temporal variations in rates of *Staphylococcus aureus* bacteremia by blood culture activity stratified by age group, Denmark, 2008–2015.

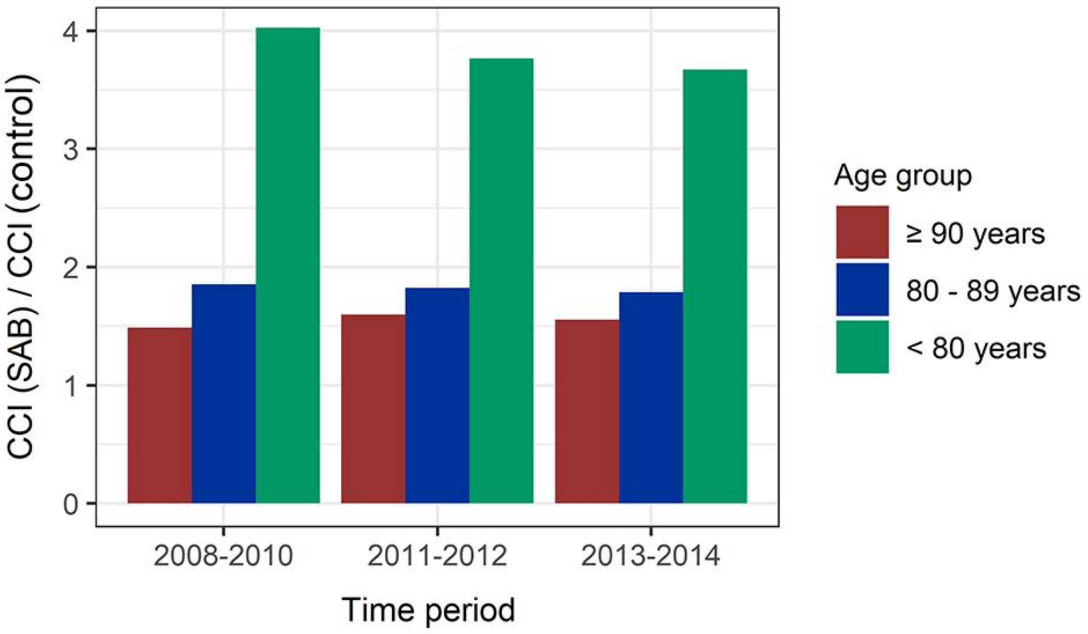

Appendix Figure 3. Relative comorbidity of *Staphylococcus aureus* bacteremia (SAB) cases and population controls stratified by 3 age groups (<80; 80–89; ≥90 years) and 3 time periods (2008–2010; 2011–2012; 2013–2014), Denmark, 2008–2015.

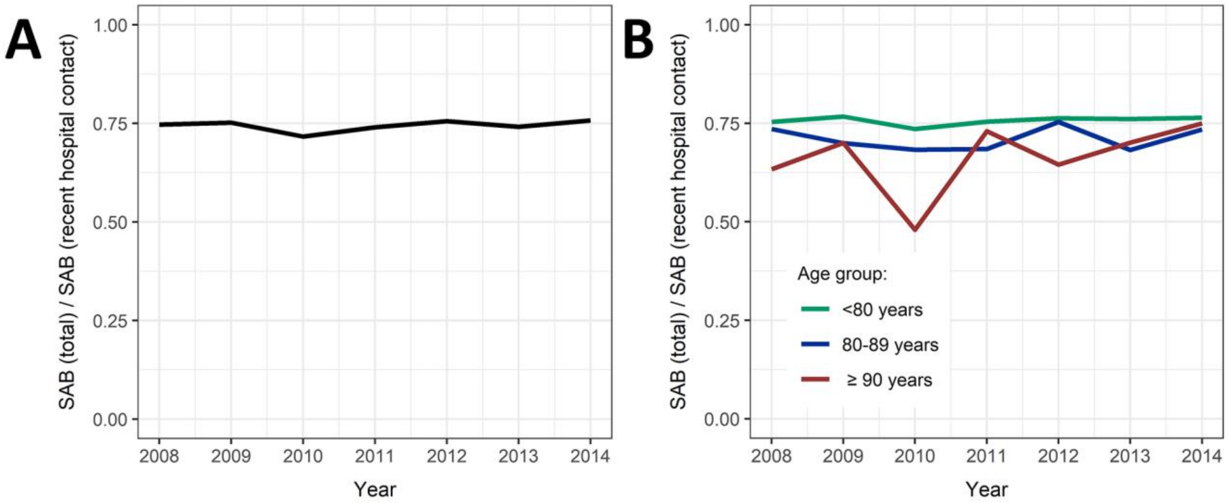

Appendix Figure 4. The proportion of *Staphylococcus aureus* bacteremia (SAB) cases with hospital contact within 90 days before SAB, Denmark, 2008–2014: A) overall and B) stratified by age groups.

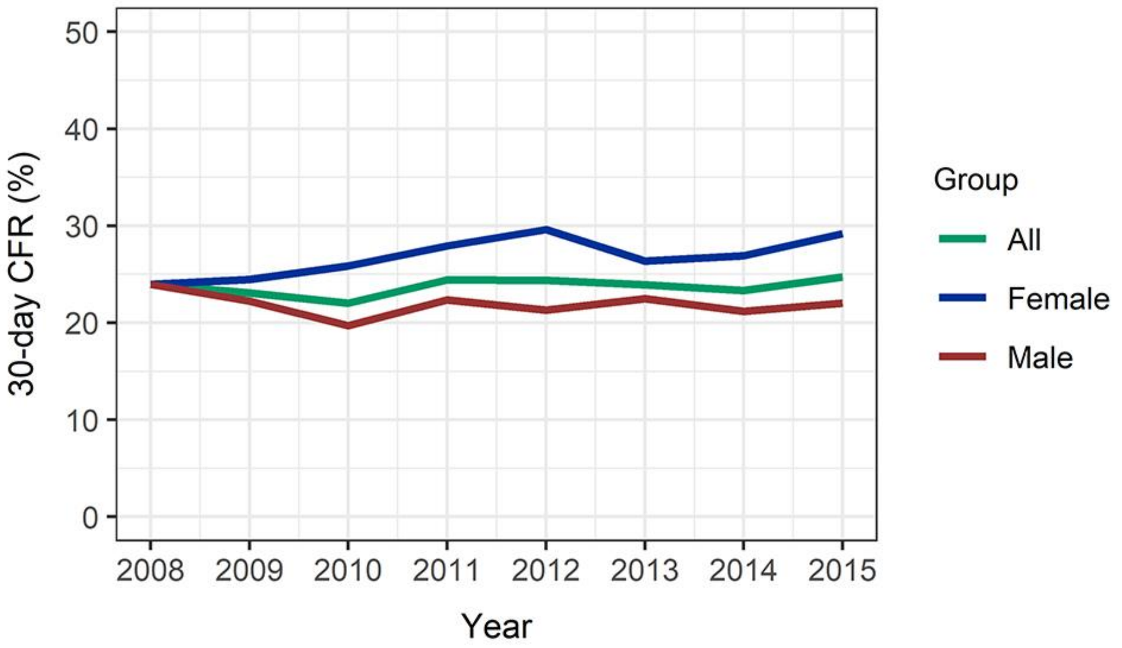

Appendix Figure 5. Thirty-day case fatality rate (CFR) from *Staphylococcus aureus* bacteremia (SAB) stratified by gender, Denmark, 2008–2015.

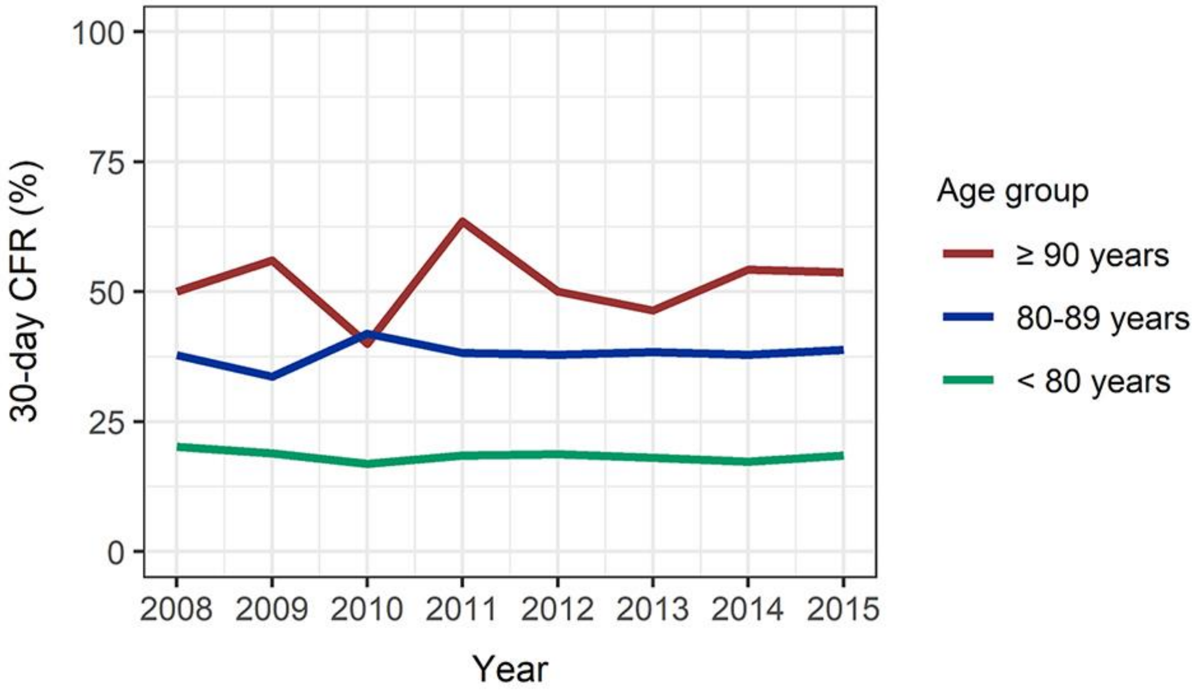

Appendix Figure 6. Thirty-day case fatality-ratio (CFR) from *Staphylococcus aureus* bacteremia (SAB) stratified by the oldest age groups, Denmark, 2008–2015.
